# A Series of Peripheral Nerve Blocks Combined With Spinal Anesthesia Is Associated With Improved Outcomes Following Total Knee Arthroplasty: A Retrospective Study

**DOI:** 10.7759/cureus.83000

**Published:** 2025-04-25

**Authors:** Kevin J Finkel, Edmund T Takata, Gregory Panza, William Stuart, Pranjali Kainkaryam, Carla L Maffeo-Mitchell, Aseel Walker

**Affiliations:** 1 Integrated Anesthesia Associates, Hartford Hospital, Hartford, USA; 2 Research Administration, Hartford Hospital, Hartford, USA

**Keywords:** anesthesia, clinical outcomes, neuraxial anesthesia, peripheral nerve blocks, spinal anesthesia, total knee arthroplasty

## Abstract

Background

Peripheral nerve blocks (PNBs) are commonly used in conjunction with general anesthesia (GA) or spinal anesthesia (SA) during total knee arthroplasty (TKA). SA is associated with reduced complications, hospital length of stay (LOS), and mortality. PNBs improve the time to discharge readiness and patient satisfaction. In 2018, we implemented a set of novel, preoperative, single-injection PNBs used with SA and intraoperative sedation. We hypothesized this regime would reduce postoperative pain, opioid use, and hospital and post-anesthesia care unit (PACU) LOS and would improve ambulation.

Materials and methods

This retrospective study compared pain scores, hospital and PACU LOS, opioid consumption, postoperative nausea and vomiting, and postoperative ambulation between two TKA patient groups: patients who received a series of preoperative PNBs and GA (PNBs-GA) and patients who received a novel series of preoperative PNBs and SA (nPNBs-SA).

Results

nPNBs-SA patients demonstrated lower average and maximum pain scores (p<0.001), shorter PACU and hospital LOS (p<0.001), and greater ambulation distance (p=0.047) compared to PNBs-GA patients. After controlling for relevant covariates, there was no significant difference in ambulation distance between the groups (p=0.519). nPNBs-SA patients demonstrated greater postoperative non-opioid use (p=0.001) and lower but not significantly different postoperative opioid use (p=0.064) compared to PNBs-GA patients.

Conclusions

Our novel series of PNBs with SA for TKA patients may reduce postoperative pain and opioid use while also improving ambulation and shortening hospital and PACU LOS.

## Introduction

Total knee arthroplasty (TKA) is one of the most common inpatient surgical procedures in the United States, with an expected annual growth rate of 7.9%. This is projected to result in approximately 3.5 million procedures by 2030. It is typically performed on elderly patients with multiple comorbidities [1-3]. Historically, postoperative pain management in TKA patients has been challenging. While a range of procedures, including general, regional, and/or neuraxial anesthetic techniques, is used in conjunction with multimodal pain regimens to manage pain and promote early mobilization, the anesthesia regimen varies significantly between clinical sites.

Neuraxial anesthesia is independently associated with lower rates of significant complications, systemic and surgical site infections, shorter hospital length of stay (LOS), and reduced mortality [2,4-8]. Notably, enhanced recovery after surgery (ERAS) programs, which include multimodal pain management strategies, are associated with reduced rates of readmission, reoperation, and mortality [9]. The addition of peripheral nerve blocks (PNBs) has also contributed to reduced time to discharge readiness and improved patient satisfaction [2].

While PNBs provide excellent analgesia in TKA, some can cause lower extremity motor weakness that is detrimental to postoperative physical therapy and can increase the risk of falls [10]. Other PNBs, such as saphenous nerve blockade in the adductor canal, provide sensory blockade while sparing motor blockade, which leads to improved postoperative analgesia and earlier postoperative ambulation when used in combination with multimodal regimens [2,11].

The development and introduction of novel anesthetic approaches for primary total hip arthroplasty and TKA are part of a concerted effort to reduce opioid use further and minimize post-anesthesia care unit (PACU) and hospital LOS [12]. In 2017, the standard anesthetic approach for TKA at our clinical center included two preoperative single-injection PNBs: infiltration of local anesthesia in the interspace between the popliteal artery and the posterior knee capsule (IPACK) and a genicular block. Patients also received a preoperative continuous-infusion saphenous nerve/adductor canal catheter that was removed on the second postoperative day, as well as intraoperative general anesthesia (GA) with muscle relaxation. In 2018, we implemented a practice change so that TKA patients would receive a novel series of preoperative single-injection PNBs, including adductor canal, IPACK, obturator, and lateral femoral cutaneous nerve (LFCN) blocks, as well as intraoperative spinal anesthesia (SA) with propofol sedation. Anecdotal evidence suggested that this change was associated with earlier ambulation, improved pain control, and a shorter hospital LOS.

This retrospective study aimed to compare pain scores, hospital and PACU LOS, opioid consumption, postoperative nausea and vomiting (PONV), and postoperative ambulation before and after the clinical center's change in perioperative anesthesia protocol for patients undergoing TKA. We hypothesized that the shift in anesthesia protocol would be associated with reduced postoperative pain, opioid use, and hospital and PACU LOS, as well as improved ambulation.

## Materials and methods

This retrospective, single-center cohort study was approved by the Institutional Review Board of Hartford HealthCare in 2019 (approval number: E-HHC-2019-0006, approval date: September 30, 2021). A waiver was granted for the requirement to obtain patient consent for collecting health information due to the minimal risk associated with the study. The participants included individuals aged over 18 and under 100 years, regardless of gender, race, or ethnicity, who underwent TKA in 2017 and were administered an adductor canal catheter along with a series of single-injection preoperative PNBs, specifically IPACK and genicular blocks, combined with GA (referred to as the PNBs-GA group). Additionally, patients who underwent TKA in 2018 received a new series of single-injection preoperative PNBs, namely IPACK, adductor canal, obturator, and LFCN blocks, alongside SA (referred to as the nPNBs-SA group). Patients were excluded from the study if they had a revision TKA, TKA performed due to trauma or fracture, underwent TKA outside the specified time frame, or did not receive any PNB.

Clinical outcomes were analyzed between the PNBs-GA group and the nPNBs-SA group. The primary outcome measures included hospital and PACU LOS, total opioid consumption, postoperative opioid use measured as morphine milligram equivalents (MME), and maximum, average, and minimum pain scores both at rest and during activity. Total opioid analgesics were collected throughout the hospitalization period, including both the intraoperative and postoperative periods. Opioid types used were hydromorphone, fentanyl, oxycodone, hydrocodone, tramadol, and morphine. These different opioid types, doses, and routes of administration were converted to MME according to an equivalency chart provided by the CDC and using the following conversion formula: (strength per unit) X (number of units/day) X (MME conversion factor) = (MME/day) [13]. Pain assessments were performed using the numeric pain scale, which assigns scores ranging from 0 to 10. This scale is commonly used in our facility to assess pain levels at rest and during activity by nurses at various intervals after surgery. Opioid medications were administered as needed when pain was not tolerable or not controlled by other scheduled non-opioid analgesics. For this study, we retrospectively collected all postoperative pain scores and determined the maximum, minimum, and average pain scores for each patient during rest and activity. We then compared these metrics between the two groups.

Secondary outcome measures included evaluating PONV, measured by counting the number of antiemetic medication doses administered after surgery, ambulation distance, and the frequency of postoperative analgesic doses. All non-opioid analgesics administered were gathered retrospectively from the electronic medical records for all patients included. These analgesics included acetaminophen and non-steroidal anti-inflammatory drugs, such as aspirin, celecoxib, diclofenac, ibuprofen, indomethacin, ketorolac, and nabumetone. Additionally, muscle relaxants such as baclofen, butalbital-APAP-caffeine, cyclobenzaprine, methocarbamol, tizanidine, gabapentin, and pregabalin were also recorded. To ensure a standardized comparison among the various types of analgesics used, we focused on the total number of doses administered (frequency) rather than the specific dosage given of each analgesic. This approach was taken because these medications were selected and calculated according to weight and overall health condition.

PNB methods

PNBs were performed preoperatively by a solo attending regional anesthesiologist, a regional anesthesia fellow, or an anesthesia resident under the direct supervision of their attending. Aseptic techniques were used, involving the preparation of the entire thigh and knee with a chlorhexidine gluconate-alcohol preparation. A 22G Stimuplex needle (B. Braun Medical Inc., Germany) was advanced under ultrasound guidance using an in-plane technique. For the complete series of blocks, a total of 80 ml of a mixture of 0.25% bupivacaine, 1:400,000 epinephrine, and 10 mg dexamethasone was injected. All injections were performed under low pressure, and negative aspirations for heme were performed after every 5 ml. Methods for the PNBs administered to PNBs-GA and nPNBs-SA patients are described below.

IPACK single-injection blocks

The popliteal artery and the posterior capsule of the knee were identified on ultrasound by placing a curvilinear ultrasound probe (FUJIFILM SonoSite, Inc., Bothell, WA) in transverse orientation at the medial aspect of the thigh approximately 1-2 fingerbreadths superior to the superior aspect of the patella. A total of 25-30 ml of the local anesthetic mixture was then injected into the space between the posterior capsule and the popliteal artery, with approximately 5 ml reserved for the most superior aspect of the posterior capsule.

Genicular nerve single-injection blocks

Ultrasound was used to locate the genicular arteries at the junction of the shaft and epicondyle of the femur and tibia; the superior medial, superior lateral, and inferior medial branches of the genicular nerves are known to travel along the genicular arteries. After negative aspiration, 1-2 ml of local anesthetic was injected under ultrasound guidance around the genicular arteries.

Adductor canal nerve single-injection blocks

The saphenous nerve was identified at the level of the adductor canal. A linear ultrasound probe (FUJIFILM SonoSite, Inc., Bothell, WA) was placed in a transverse orientation at the anteromedial thigh at the level of mid-thigh, with identification of the sartorius muscle forming the roof of the canal and the adductor longus muscle forming the floor of the canal. The superficial femoral artery and vein were identified. Lateral to these structures and outside of the canal, yet deep to the sartorius, we identified the nerve to vastus medialis, and its location was confirmed using nerve stimulation. After confirmation, 10 ml of local anesthetic solution was deposited to surround the nerve to the vastus medialis, and 20-25 ml of the solution was then injected into the adductor canal to bathe the saphenous nerve. The saphenous nerve block was confirmed by observing the characteristic compression of the superficial femoral artery and vein as the local anesthetic was injected into the canal.

Adductor canal catheter continuous-injection blocks

A local anesthetic mix was injected in the proximal-to-central part of the adductor canal, and a nerve catheter was threaded into the canal around the superficial femoral artery. The nerve catheter was secured with tape, and an infusion of 0.2% ropivacaine was started in the PACU at a rate of 10 ml per hour and continued until postoperative day 2.

Obturator nerve single-injection blocks

We identified the interfascial spaces in which the anterior and posterior divisions of the obturator nerve lie. The linear ultrasound probe was placed in a transverse orientation at the level of the anterior inferior iliac spine and then translated medially until the femoral vessels were visualized. The probe was then translated medially to identify the three muscle layers: adductor longus, most superiorly; adductor brevis; and adductor magnus, most inferiorly. Identification of the plane containing the anterior division of the obturator nerve was confirmed using nerve stimulation at the interfascial plane between the adductor longus and the adductor brevis, and identification of the plane containing the posterior division of the obturator nerve was confirmed using nerve stimulation at the interfascial plane between the adductor brevis and adductor magnus. After confirmation, 10 ml of local anesthetic mixture was injected into each plane.

LFCN single-injection blocks

The LFCN was identified using a linear ultrasound probe in a transverse orientation at the level of the superior thigh, just inferior to the anterior inferior iliac spine. After performance of the obturator block, the probe was translated laterally, and the sartorius was followed until its most lateral edge was obtained. The LFCN was often identified at this location between the sartorius muscle and the tensor fasciae latae muscle. However, confirmation of its variable placement was provided by nerve stimulation, which elicited a “tapping” sensation on the lateral aspect of the patient's thigh. With confirmation, the final 5 ml of local anesthetic solution was injected into the space.

Data management and sample size calculation

Data were retrieved from an electronic medical record system. Primary identifiers were removed, and a unique study identification number was assigned to each patient. Medical record numbers and dates of service were collected and used to create a database containing only de-identified information. Data was stored electronically on a server that was only accessible by the research team. We aimed to include all eligible patient records from 2017 and 2018 that met the inclusion criteria to maximize the study sample size. The included sample of 789 patient charts, with a 64% (PNBs-GA) to 36% (nPNBs-SA) group allocation, was sufficient to detect a minimum effect size of 0.208 (small) at 80% power for the primary outcomes of interest, including pain scores and LOS.

Statistical analyses

All data were checked for normality. Primary analyses compared the following clinical outcomes between the two groups: hospital and PACU LOS, opioid use, pain scores, PONV scores, and ambulation. Categorical variables were examined using the chi-square test. Continuous variables were analyzed using the independent samples t-test when the data were normally distributed and the Mann-Whitney U test when the data were not normally distributed.

Multivariate regression analyses of the entire dataset were used to investigate the association between postoperative opioid use and over-the-counter medications while controlling for relevant covariates (e.g., history of psychiatric disorder and chronic pain). A multivariate regression analysis was performed to examine the association between postoperative opioid use and pain scores while controlling for the same covariates. An alpha level of 0.05 was deemed statistically significant. Statistical analyses were performed using SPSS Statistics version 26.0 (IBM Corp. Released 2019. IBM SPSS Statistics for Windows, Version 26.0. Armonk, NY: IBM Corp.).

## Results

A total of 789 patients who underwent TKA at our institution between 2017 and 2018 were included in the analysis. Of these patients, 282 (35.7%) were in the PNBs-GA group and 507 (64.3%) were in the nPNBs-SA group.

Patient demographics and clinical characteristics at baseline for each group are presented in Table 1. In the PNBs-GA group, there were significantly fewer White patients and more Asian patients (p=0.043) as well as a significantly higher body mass index (p=0.014). There were no other significant differences in baseline characteristics between the two groups.

**Table 1:** Baseline demographics and clinical characteristics compared between PNBs-GA and nPNBs-SA patients Data are reported in frequency (proportion) unless stated otherwise. Variables with data presented as means (SD) and t-statistics were compared using an independent samples t-test. Variables with data presented as frequencies (%) and X² statistics were compared using chi-square analysis. * significant differences (p<0.05) between groups with Bonferroni correction for multiple comparisons. SD: standard deviation, ASA: American Society of Anesthesiologists, PNBs-GA: series of preoperative peripheral nerve blocks and general anesthesia, nPNBs-SA: novel series of preoperative peripheral nerve blocks and spinal anesthesia

Variable	PNBs-GA (n=282)	nPNBs-SA (n=507)	Both groups (N=789)	Statistic	p-value
Age, year, mean (SD)	67.2 (9.0)	67.9 (8.9)	67.6 (8.9)	-1.04	0.297
Sex, male	107 (37.9)	186 (36.7)	293 (37.1)	0.12	0.726
Race				8.14	0.043
White or Caucasian	231 (81.9)	445 (87.8)	676 (85.7)*		
Black or African American	23 (8.2)	33 (6.5)	56 (7.1)		
Asian	4 (1.4)*	1 (0.2)	5 (0.6)		
Other	24 (8.5)	28 (5.5)	52 (6.6)		
Body mass index, kg/m^2^, mean (SD)	33.0 (7.4)	31.8 (6.0)	32.2 (6.5)	2.33	0.014
Total comorbidities, mean (SD)	1.9 (1.8)	1.9 (1.9)	1.9 (1.8)	0.20	0.843
ASA physical status				3.84	0.280
ASA-I	1 (0.4)	6 (1.2)	7 (0.9)		
ASA-I	175 (62.1)	339 (66.9)	514 (65.1)		
ASA-III	105 (37.2)	161 (31.8)	266 (33.7)		
ASA-IV	1 (0.4)	1 (0.2)	2 (0.3)		

**Figure 1:**
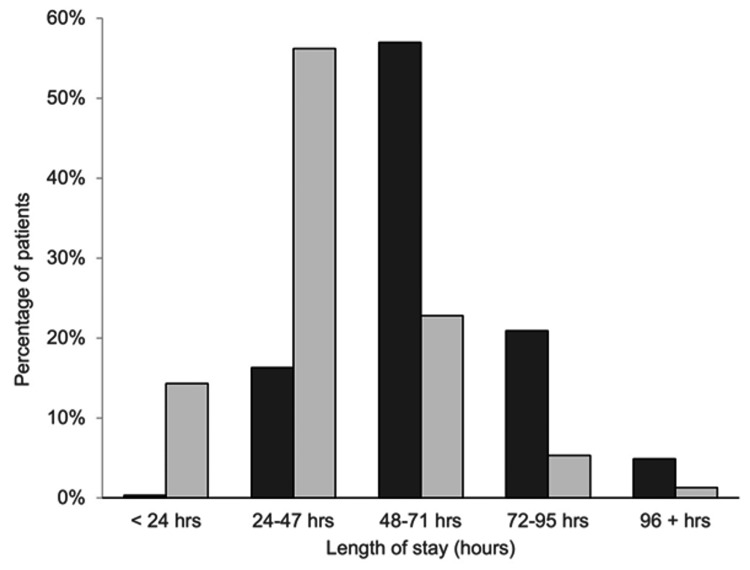
Distribution of hospital LOS for PNBs-GA (⬛) and nPNBs-SA (⬜) patients following TKA. PNBs-GA patients underwent TKA during 2017 and received an adductor canal catheter, a series of single-injection, preoperative PNBs (IPACK and genicular blocks), and GA. nPNBs-SA patients underwent TKA during 2018 and received a novel series of preoperative PNBs (IPACK, adductor canal, obturator, and LFCN blocks) and SA intraoperatively LOS: length of stay, TKA: total knee arthroplasty, GA: general anesthesia, PNB: peripheral nerve block, SA: spinal anesthesia, IPACK: interspace between the popliteal artery and capsule of the posterior knee, LFCN: lateral femoral cutaneous nerve, PNBs-GA: series of preoperative peripheral nerve blocks and general anesthesia, nPNBs-SA: novel series of preoperative peripheral nerve blocks and spinal anesthesia

Pain scores at rest and during activity are presented in Table 2, and hospital and PACU LOS are shown in Table 3, with the distribution of hospital LOS shown in Figure 1. Maximum pain at rest, average pain at rest, maximum pain with activity, and average pain with activity were significantly greater in the PNBs-GA group (p<0.001). Minimum pain at rest and minimum pain with activity were not significantly different between groups. Median hospital and PACU LOS were significantly longer in the PNBs-GA group (p<0.001), while median ambulated distance was significantly longer in the nPNBs-SA group (Table 3; p=0.047).

**Table 2:** Pain scores at rest and during activity compared between PNBs-GA and nPNBs-SA patients Data are reported as mean (SD). Groups were compared using an independent samples t-test. ^a ^Sample sizes for pain scores "at rest": PNBs-GA (n=281), nPNBs-SA (n=501), and both groups (N=782). Sample sizes for pain scores "with activity": PNBs-GA (n=278), nPNBs-SA (n=493), and both groups (N=771). SD: standard deviation, PNBs-GA: series of preoperative peripheral nerve blocks and general anesthesia, nPNBs-SA: novel series of preoperative peripheral nerve blocks and spinal anesthesia

Variable	PNBs-GA^a^ (n=282)	nPNBs-SA^a^ (n=507)	Both groups^a^ (N=789)	t-statistic	p-value
Minimum pain at rest	1.0 (1.5)	1.1 (1.5)	1.1 (1.5)	-0.10	0.922
Maximum pain at rest	7.9 (1.7)	6.1 (2.4)	6.8 (2.3)	11.89	<0.001
Average pain at rest	4.4 (1.5)	3.5 (1.7)	3.8 (1.7)	8.23	<0.001
Minimum pain with activity	2.4 (2.2)	2.4 (2.4)	2.4 (2.3)	0.29	0.780
Maximum pain with activity	7.9 (1.8)	6.5 (2.4)	7.0 (2.3)	9.52	<0.001
Average pain with activity	5.4 (1.7)	4.5 (2.1)	4.8 (2.0)	6.61	<0.001

**Table 3:** Hospital and PACU LOS and postoperative ambulated distance compared between PNBs-GA and nPNBs-SA patients Data are reported as median (interquartile range). Groups were compared using the Mann-Whitney U test. PACU: post-anesthesia care unit, LOS: length of stay, PNBs-GA: series of preoperative peripheral nerve blocks and general anesthesia, nPNBs-SA: novel series of preoperative peripheral nerve blocks and spinal anesthesia

Variable	PNBs-GA (n=282)	nPNBs-SA (n=507)	Both groups (N=789)	U-statistic	p-value
Hospital LOS, hours	53.3 (48.8-72.1)	29.6 (25.5-48.9)	47.5 (27.8-53.9)	23974.50	<0.001
PACU LOS, minutes	143 (113-191)	105 (81-139)	119 (89-157)	41027.00	<0.001
Ambulated distance, feet	300 (185-470)	350 (250-450)	325 (235-450)	61589.50	0.047

The use of postoperative opioid, non-opioid, and antiemetic medications is presented in Table 4. While the median number of postoperative doses of non-opioid pain medication was significantly greater in the nPNBs-SA group (p<0.001), total MME, postoperative MME, the use of antiemetic medication, and the frequency of chronic opioid users were not significantly different between groups.

**Table 4:** Use of antiemetics, opioids, and non-opioid pain medication compared between PNBs-GA and nPNBs-SA patients Data reported in median (interquartile range) with U-statistic were compared using the Mann-Whitney U test. Chronic opioid users were compared between groups using chi-square analysis and presented as frequency (proportion) with X^2^ statistic. MME: morphine milligram equivalents, PNBs-GA: series of preoperative peripheral nerve blocks and general anesthesia, nPNBs-SA: novel series of preoperative peripheral nerve blocks and spinal anesthesia

Variable	PNBs-GA (n=282)	nPNBs-SA (n=507)	Both groups (N=789)	Statistic	p-value
Opioid use, MME					
Total	106 (52-196)	95 (40-171)	98 (44-176)	66022.00	0.075
Postoperative	92 (40-168)	80 (30-151)	83 (32-157)	65816.50	0.064
Doses of postoperative non-opioid analgesics administered	18 (13-24)	20 (15-27)	19 (14-26)	61446.50	0.001
Doses of antiemetics administered	0 (0-1)	0 (0-1)	0 (0-1)	6884.50	0.292
Chronic opioid users	35 (12.4)	84 (16.6)	119 (15.1)	2.45	0.118

The results of the multivariate analysis of the associations between group, hospital, PACU LOS, and ambulation are shown in Table 5. After controlling for gender, age, race, body mass index, American Society of Anesthesiologists rating, and total comorbidities, patients in the nPNBs-SA group were associated with decreased average and maximum pain during rest and activity, shorter hospital and PACU LOS, and increased ambulation distance. Additional multivariate analyses found no association between postoperative opioid use and pain scores, including minimum, maximum, and average scores both with rest and with activity (p>0.05). However, the number of anti-inflammatory analgesic medication prescriptions was associated with an increase in postoperative opioid use (unstandardized regression coefficient beta (USC B)=31.97, 95% confidence interval (CI)=6.64, 57.30, p=0.013). There was also a trend towards an increase in postoperative opioid use in patients who were chronic opioid users (USC B=27.70; 95% CI=-4.16, 59.55; p=0.088). No other home medications (including over-the-counter and prescription medications) that were taken outside of the admission period were associated with postoperative opioid use.

**Table 5:** Association of "treatment group" with hospital and PACU LOS, pain with rest and activity, and ambulation distance while controlling for relevant covariates Covariates included age, gender, race, body mass index, ASA rating, and total comorbidities. PNBs-GA was used as the reference value for the "treatment group" variable. PACU: post-anesthesia care unit, LOS: length of stay, USC B: unstandardized regression coefficient beta, SE: standard error, CI: confidence interval, ASA: American Society of Anesthesiologists, PNBs-GA: series of preoperative peripheral nerve blocks and general anesthesia, nPNBs-SA: novel series of preoperative peripheral nerve blocks and spinal anesthesia

Variable	USC B	SE	Lower 95% CI	Upper 95% CI	p-alue
Hospital LOS, hours	-22.721	1.469	-25.605	-19.838	<0.001
PACU LOS, hours	-41.717	4.136	-49.836	-33.599	<0.001
Average pain at rest	-0.921	0.119	-1.154	-0.688	<0.001
Average pain with activity	-0.883	0.144	-1.166	-0.600	<0.001
Maximum pain at rest	-1.723	0.160	-2.036	-1.409	<0.001
Maximum pain with activity	-1.412	0.162	-1.731	-1.094	<0.001
Minimum pain at rest	0.039	0.111	-0.180	0.257	0.728
Minimum pain with activity	-0.028	0.174	-0.370	0.314	0.874
Ambulation distance, feet	12.245	18.969	-24.993	49.482	0.519

## Discussion

Our retrospective study addressed the lack of conclusive data describing the relationship between anesthetic technique and clinical outcomes in TKA patients. Specifically, we examined two cohorts of patients who underwent TKA before or after the introduction of a new anesthesia regimen for TKA at our clinical center. Compared with the previous use of an adductor canal catheter, a series of PNBs, and GA at our institution for TKA patients, the implementation of a novel series of PNBs and SA was associated with a significant improvement in pain scores at rest and during activity, postoperative ambulated distance, and hospital and PACU LOS. Opioid use did not significantly change after the new regime, yet postoperative non-opioid use significantly increased. The clinical implications of our findings include that the novel series of PNBs implemented alongside SA studied here may be effective in reducing postoperative pain, improving postoperative ambulation, and shortening hospital and PACU LOS at similar institutions for patients undergoing TKA. It adds to the evidence that SA and PNBs can effectively be implemented for postoperative pain management in TKA. Thus, it supports the continued trial of similar pain control regimens that may achieve similar improvements in patient clinical outcomes.

Successful TKA typically results in greater mobility, reduced pain, and an improved quality of life [1]. However, recovery from TKA is often more challenging and painful than other orthopedic procedures. Furthermore, consistent participation in a physical therapy program is recommended for patients to optimize functional recovery with their replacement joint [14]. Effective pain management and mobility are vitally important for optimizing patient outcomes [14]. When postoperative pain is optimally managed, TKA patients often experience a shorter hospital LOS and improvements in mobility and ambulation, independent of physical therapy [15].

To effectively combat the continued opioid crisis, it is also important that refinements to anesthetic techniques for TKA aim to optimize pain control, enhance functional rehabilitation, and improve overall patient satisfaction while having the overall goal of limiting opioid use. PNBs and central neuraxial blockade are widely used techniques that effectively reduce postoperative pain and opioid consumption [10,16,17]. These nerve blocks and other modern anesthetic strategies, such as intraoperative periarticular injections, continue to have a positive impact on postoperative pain management in TKA [2,5,12]. Since both peripheral and central mechanisms are involved in postoperative pain in TKA, PNBs, neuraxial anesthesia, or local infiltration analgesia alone are unable to provide satisfactory pain relief [18]. Multimodal analgesia regimens target pain both centrally and peripherally by combining these anesthesia approaches with additional perioperative strategies, such as patient-controlled analgesia, oral analgesic medications (including opioids and non-opioids), and preoperative and postoperative administration of a combination of acetaminophen, gabapentinoids, and cyclooxygenase-2 inhibitors, to provide adequate pain relief. Several clinical studies have shown that multimodal analgesia provides superior pain relief after TKA [19-21]. Now considered the optimal strategy for managing pain in TKA patients [18], multimodal analgesia mimics the success of ERAS protocols established for other patient populations.

This study has several limitations. First, it is a retrospective, single-site study and included patients who received treatment as early as 2017. The sample size for retrospective studies is limited to the number of patient charts that meet inclusion criteria within the chart inclusion timeframe. The actual number of patient charts was lower than initially expected, based on pre-data extraction estimates. However, our sample was adequate to detect small effects in our primary endpoints of interest. While this study aimed to examine the impact of a change in block anesthesia protocols that occurred in 2018, multimodal analgesia pain management protocols were also implemented during the study period. While we attempted to control for this confounding factor using multivariate regression analyses, ideally, the use of multimodal analgesia would have been universal across both study groups. Second, changes in PNB technique in the comparison groups and the transition from GA to SA during intraoperative procedures occurred during the same time period. SA is known to be associated with improved pain scores and lower opioid use following TKA [22]; therefore, the positive changes observed following the practice change may be attributed to effects from the novel PNBs, SA, or a combination of both, in addition to other unmeasured changes that may have occurred over this period. Additionally, in an attempt to determine patients who were chronic opioid users, we used medication prescription history as a proxy, which may not have reflected actual opioid consumption and, therefore, may have led to inaccuracies regarding this variable. Furthermore, quantifying the use of non-opioid analgesics was challenging since, unlike opioid analgesics, non-opioid analgesics do not have a standard metric to quantify their analgesic effect. Consequently, we standardized the use of non-opioid analgesic medications by assuming a single dose of any medication was identical. In addition, body mass index was significantly different between groups, and opioid use per kilogram, which may have been a more appropriate comparison given this difference, was not compared between groups. Finally, PONV was estimated based on antiemetic consumption, as data from more direct sources, such as surveys, were not available.

## Conclusions

This retrospective cohort study of preoperative PNB techniques combined with SA showed improved rehabilitation after TKA, characterized by reduced pain, shorter hospital and PACU LOS, and better recovery of mobility. As TKA will likely continue to be one of the most frequently performed procedures, optimizing the pain management and rehabilitation protocols following surgery is critical. Therefore, larger, prospective studies should further explore how the interventions examined in this study, which included a novel PNB series plus SA protocol, might improve the outcomes of TKA in the future.
